# Antioxidant and Antineoplastic Activities of *Hibiscus sabdariffa* Linn. Petal Extracts against Oral Squamous Cell Carcinoma Cell Line

**DOI:** 10.3290/j.ohpd.b4997059

**Published:** 2024-02-20

**Authors:** Hadeel Mazin Akram, Azhar M. Haleem, Rasha Salah

**Affiliations:** a Assistant Professor, Department of Periodontics, University of Baghdad /College of Dentistry, Baghdad, Iraq.; b Assistant Professor, Environment Research Center/University of Technology, Baghdad, Iraq.; c Lecturer, Department of Periodontics, University of Baghdad /College of Dentistry, Baghdad, Iraq.

**Keywords:** DPPH, *Hibiscus sabdariffa*, methanol, natural anticancer compound, squamous cell carcinoma

## Abstract

**Purpose:**

To assess the antioxidant and antineoplastic effects of *Hibiscus sabdariffa* Linn. on oral squamous cell carcinoma cells.

**Materials and Methods:**

Human squamous cell carcinoma HSCC cells were tested for cytotoxicity by a methanol extract of *Hibiscus sabdariffa* (MEHSP). After 24, 48, and 72 h, the MTT assay and Trypan blue exclusion test were used to determine cell survival and death. 2, 2-diphenyl-1-picrylhydrazyl (DPPH), DNA Protection Assay (DPA), and ferric reducing antioxidant power assay (FRAPA) measured the antioxidant activity of MEHSP.

**Results:**

The antioxidant activity (%) ranged from 47.92-82.24 in the DPPH test, 11.61-73.65 in the DPA, and 4.97-52.09 in the FRAPA. The HSCC in-vitro cytotoxicity assay showed dose- and time-dependent cell viability. MEHSP at 5 μg/ml inhibited viable cells, while increasing MEHSP doses decreased cell viability. The Trypan blue exclusion test showed that MEHSP significantly reduced cell viability at 24, 48, and 72 h.

**Conclusion:**

*Hibiscus sabdariffa* contains antioxidant and HSCC-cytotoxic properties.

Human oral squamous cell carcinoma (HOSCC) has a high incidence rate globally, ranking sixth among all types of cancers. In fact, it has been reported that squamous cell carcinoma contributes to 90% of all oral tumors.^32^ The invasive potential of tumor cells was discovered to be connected with worse outcome of oral squamous cell carcinoma,^23^ a multi-factorial disease.^25^ Smoking, betel quid use, alcohol consumption, HPV infection, genetics, radiation, a poor diet, and lack of physical exercise are just some of the risk factors for HOSCC.^11^ Dysbiosis can promote different oral diseases, including periodontitis. It has also been associated with cancer.^22^ Oral squamous cell carcinomas have a varied microbiome that differs from healthy and precancerous tissues. Emerging trends link periodontal bacteria such as *Porphyromonas gingivalis* and *Fusobacterium nucleatum* to cancer.^1,14^ The 5-year survival rate is 50%. Early detection increases this rate up to 60%-80%.^27^ Up to now, the main means of treatment have been surgery and radiotherapy. However, these methods can be harmful despite recent improvements, as they cause anemia, loss of appetite, or peripheral neuropathy. All these conditions affect the individual’s general well-being. This therefore necessitates the development of cancer treatments with less severe side-effects.^5^ The use of alternative medicine is growing worldwide, particularly concerning the consumption of plant-based substances. This increase can be attributed to the fact that herbal remedies are less damaging and more compatible with our bodies, compared to synthetic medications, which makes them appropriate for long-term use.^12^ Antioxidant therapy is a relatively new method for combating cancer. DNA and the intracellular structure of cells are compromised by oxidative stress. Oxidative stress reduction can be accomplished using antioxidants that scavenge free radicals.^28^ Some bioactive compounds found in most plants have been investigated and found to be advantageous for human health.^4,31^ *Hibiscus sabdariffa* Linn. (family: Malvaceae), commonly known as Roselle or Sorrel,^24,26,27^ is a well-known dicotyledonous, annual or perennial herb or woody subshrub found in Asia and Africa. Its uses span the food, beverage, and healthcare industries, making it a valuable plant.^8^ Leaves, seeds, stems, and calyces are all edible and flavourful plant elements that can be included in food and herbal drinks.^18^ *Hibiscus sabdariffa* tea has been reported to be relatively safe even when consumed in high concentrations.^16^ For centuries, beverages made from the calyces of *Hibiscus sabdariffa* have been utilised in traditional medicine to address various ailments, e.g., cough, hypertension, ulcers, heart diseases, gastrointestinal disorders, urinary issues, fever, and even cancer.^21^ The calyces of *Hibiscus sabdariffa* possess a variety of active compounds, such as alkaloids, anthocyanins, phenols, saponin, tannin, and flavonoids.^20^ Cancer and other diseases have been effectively treated and prevented with the use of key elements found in *Hibiscus sabdariffa*.^6^ It was found that the calyces of *Hibiscus sabdariffa* are rich in anthocyanins and have metabolic and hepatoprotective properties.^9,30^ A study showed that consumption of flavonoid-rich foods could protect effectively against the occurrence and progression of different cancer types.^28^ Consumption of flavonoids may reduce the risk of pharyngeal, laryngeal and esophageal cancers by 70%.^4^ The mortality rate among individuals who take in a considerable amount of soy isoflavones is lower than in those who do not.^9^ Flavonoids may suppress invasion, metastasis, angiogenesis and apoptosis, thus inhibiting cancer progression.^1^ The aim of this work is to evaluate the antioxidant and antineoplastic properties of *Hibiscus sabdariffa* Linn. in relation to a squamous cell carcinoma cell line.

## Materials and Methods

### Plant Extracts

Dry petals of *H. sabdariffa* (FGO Organics; Seattle, WA, USA) were purchased from local markets in Baghdad, Iraq. They are labeled as harvested in Egypt, and were identified by a taxonomist at the College of Science, University of Baghdad.

An electric grinder was used to break up the dry petals of *H. sabdariffa*. Then, 250 g of the fine powder was measured and put in 500 ml of 100% methanol at room temperature with constant stirring for 7 days. After that, Whatman No. 1 filter paper was used to separate the mixture. The crude extract was put in a rotating evaporator to remove the solvent and evaporated under vacuum pressure at 3000 rev/min at 45°C. The concentrate from the rotating evaporator was dried until there was no change in mass after more drying time, and a 7-g methanol extract of *H. sabdariffa* petals (called MEHSP) was made.

### DPPH Radical-scavenging Assay

The methodology developed by Moraes-de-Souza et al^19^ was followed to assess the DPPH radical-scavenging activity of MEHSP, after making the necessary adjustments. A series of extracts – 0.0, 5, 10, 25, 50, 125, 250 and 500 µg/ml – using a methanol solution of MEHSP were prepared; 0.1 millimolar of 2, 2-diphenyl-1-picrylhydrazyl (DPPH) was added and vortexed. After an incubation period of 60 min at room temperature, the absorbance was determined using an ELx800 microplate reader (Elisa Reader – Dana 3200, Prestige Diagnostics; Antrim, Northern Ireland, UK). The reading was taken at 517 nm. To determine the percentage of antioxidant activity (%AA), the following equation was used:


$$\begin{array}{ccc} \frac{\text{Absorbence of sample}}{\text{Absorbence of standard}}\times 100-100 & & \left(1\right) \end{array}$$

### DNA Protection Assay (DPA)

The protective ability of MEHSP on pure DNA molecules was quantified using the spectrophotometric method, using pure DNA from fish sperm, which has a 260:280 absorbance ratio (1.87). The method involved mixing identical volumes of DNA solution and MEHSP at different concentrations (0.0, 5, 10, 25, 50, 125, 250 and 500 µg/ml), after treating it with a 1x10^-5^ M H_2_O_2_ solution. This mixture was incubated for 10 min at 37°C. Following the incubation period, the absorbency was recorded at 260 nm and the protection percent was determined by applying the following equation:


$$\begin{array}{ccc} \frac{\text{Absorbence of sample}}{\text{Absorbence of standard}}\times 100-100 & & \left(2\right) \end{array}$$

### Ferric-reducing Antioxidant Power Assay

The method described by Dinis et al^7^ was used. A series of different MEHSP concentrations – 0.0, 5, 10, 25, 50, 125, 250 and 500 µg/ml – was prepared. The reducing power of ferrous ions (Fe^2+^) was measured for the prepared extracts by adding 100 µl of the test substance to 3 ml of methanol and 1 ml of 2 mM of ferrous chloride (FeCl_2_). After 10 min of incubation at ambient temperature, the absorbance was calculated at 562 nm.

The following equation was used to compute the antioxidant activity percent (AA%):


$$\begin{array}{ccc} \frac{\text{Absorbence of sample}}{\text{Absorbence of standard}}\times 100-100 & & \left(3\right) \end{array}$$

### MTT Assay

The HSCC line was grown in a medium containing 10% fetal calf serum (FCS) following the recommendations of the manufacturer (Sigma Aldrich; St Louis, MO, USA). Penicillin and streptomycin solutions were added to the medium at concentrations of 50 IU/ml and 500 g/ml, respectively. The cells were kept at 37°C with 5% CO_2_ and 95% humidity. To prepare the MEHSP solution for experimentation, it was dissolved in phosphate buffered saline (PBS) and diluted to working concentrations of 0.0, 5, 10, 25, 50, 125, 250, and 500 g/ml. This solution was directly added to the culture medium after seeding 100,000 HSCC cells per well in microplates with a capacity of up to 96 wells. The mixture was then incubated for a period of 12 h at a temperature of 37°C with air and a CO_2_ concentration of around 5%. The MTT assay was performed by adding quantities (5, 10, 25, 50, 125, 250 and 500 g/ml) of extracts to the microplates which were then further incubated for 24, 48, and 72 h under the same conditions. Each microplate had one column that served as a negative control, devoid of any extract. The excess culture medium was taken out, and each well was then treated with a solution of 3-(4, 5-dimethylthiazol- 2-yl)-2, 5-diphenyltetrazolium bromide (MTT) at a final concentration of 0.5 mg/ml medium for 4 h at 37°C. After the incubation period, formazan crystals were formed, which can be quantified by dissolving them in absolute ethanol. The absorbance of each well was measured spectrophotometrically at 560 nm in a microplate reader (Elisa Reader – Dana 3200). The impact of MEHSP on HSCC viability was determined using non-exposed cells’ absorbance.

### Trypan Blue Vital Staining

Trypan blue, a dye that differentiates between living and dead cells, was used to determine the percentage of deceased cells. After being treated with MEHSP for 24, 48, and 72 h, HSCC cells were placed at a density of 100,000 cells/ml in a dish with a diameter of 60 mm. Equal amounts of cell suspension and 1% Trypan blue were used to collect the adhering and floating cells, which were then washed twice in PBS and then stained. Following the incubation period (3 min at 37°C), the mixture was examined under a microscope. Trypan blue-stained cells were counted as dead. The viability percent was calculated according to the following equation:


$$\begin{array}{ccc} \text{Viability }\%=\frac{\text{Non-stained cells}}{\text{total cells }(\text{stained}+\text{non-stained})}\times 100 & & \left(4\right) \end{array}$$

### Cytogenetic Analysis

Peripheral blood cells were pre-stimulated with 10 µg/ml phytohaemagglutnine (PHA) and incubated for 72 h at 37ºC and 5% CO_2_ ambient air. Whole blood was drawn from a healthy 25-year-old man using a sterile syringe coated with heparin. 0.5 ml of blood was added to 4.5 ml of completed culture medium RPMI-1640, which was enriched with 10% fetal bovine serum and 10 µg/ml of antibiotics (penicillin and streptomycin). Subsequently, the cultured cells were subjected to 10 µg/ml colchicine for a duration of 20 min, followed by a 20-min exposure to hypotonic KCl 0.075M. Following a 5-min centrifugation at 3000 rpm, the pellet underwent three washings with a fixative solution (3:1 methanol:glacial acetic acid). The supernatant was discarded. After the clear cell solution was allowed to drop on clean, cold slides for an entire night, it was stained with Giemsa stain. Chromosomal aberrations, blastogenic index, and mitotic index were measured for both exposed and non-exposed cells at varying doses of the MEHSP.^31^

### Statistical Analysis

The data were analysed using SPSS (version 23, IBM; Armonk, NY, USA). Least Significant Differences (LSD) and one-way ANOVA were used to determine statistically significant differences with the p-value set at < 0.05. Means and standard deviations were used to describe the collected data.

## Results

### Antioxidant Activity Assay

The scavenging activity of DPPH, DPA, and FRAPA of MEHSP increased with increasing concentrations. As shown in Tables 1 – 3, the antioxidant activity (%) ranged from 47.92-82.24 in the DPPH assay (p = 0.021), from 11.61 to 73.65 in the DPA assay (p = 0.044), and the antioxidant percentage for FRAPA ranged from 4.97 to 52.09 (p = 0.03). All differences were statistically significant.

**Table 1:** Antioxidant capacity of different concentrations of MEHSP by DPPH method

Concentration µg/ml	Abs	AA%
0.0	0.553 ± 0.01	0.0
5	0.288 ± 0.03*	47.92
10	0.276 ± 0.03*	50.09
25	0.245 ± 0.02*	55.69
50	0.232 ± 0.01*	58.04
125	0.211 ± 0.01*	61.84
250	0.118 ± 0.03*	78.66
500	0.092 ± 0.01*	82.24
1% ascorbic acid	0.099 ± 0.01*	82.09

p = 0.021. Each number represents mean for three replicates. *Significant at p < 0.05.

**Table 2:** Antioxidant capacity of different concentrations of MEHSP by DNA protective method

H_2_O_2_		
Concentration µg/ml	Abs	AA%
0.0	0.482 ± 0.010	0.0
5	0.426 ± 0.021*	11.61
10	0.386 ± 0.011*	19.91
25	0.319 ± 0.02*	33.81
50	0.288 ± 0.011*	40.24
125	0.212 ± 0.012*	56.01
250	0.155 ± 0.011*	67.84
500	0.127 ± 0.013*	73.65
DNA + H_2_O_2_ (1×10^-5^) M	0.482 ± 0.011	
DNA	0.266 ± 0.012*	

p = 0.044. Each number represents mean ± SD of three replicates. *Significant at p < 0.05.

**Table 3:** Antioxidant capacity of different concentrations of MEHSP by ferric reducing method

FeCl_3_		
Concentration µg/ml	Abs	AA%
0.0	0.382 ± 0.012	0.0
5	0.363 ± 0.013*	4.97
10	0.324 ± 0.021*	15.18
25	0.301 ± 0.011*	21.20
50	0.288 ± 0.022*	24.60
125	0.245 ± 0.011*	35.86
250	0.211 ± 0.014*	44.76
500	0.183 ± 0.010*	52.09
1% ascorbic acid	0.160 ± 0.011*	58.11

p = 0.031. Each number represents mean of three replicates. *Significant at p < 0.05.

### Suppression of HOSCC cells by MEHSP (In Vitro)

**Fig 1:**
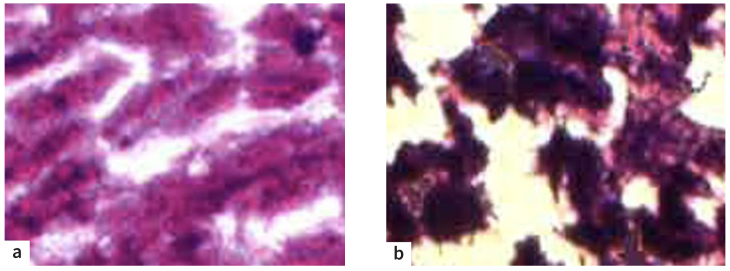
a. Non-exposed HSCC cell line (control) exhibits cohesive malignant cells in a confluent monolayer with no empty spaces. b. Exposed to 25 µg/ml of methanol extract of *Hibiscus sabdariffa*. H&E stain; 400X magnification.

The results of the in-vitro cytotoxicity test on HOSCC are shown in Tables 4 and 5, which demonstrate that cell viability is dose- and time-dependent. The experiment revealed that the MEHSP concentration of 5 μg/ml resulted in suppression of viable cells. Furthermore, it was observed that the decrease in cell viability became more pronounced as the concentration of MEHSP increased. The Trypan blue exclusion test showed that MEHSP caused a statistically significant drop in cell viability for each exposure time (24, 48, and 72 h), as shown in Table 6. Furthermore, Fig 1 demonstrates the toxic effects of MEHSP on the vitality of HOSCC by changing the morphological features. The control groups exhibited a completely continuous layer of cohesive malignant cells that displayed pleomorphism, hyperchromatism, well differentiation, and a high ratio of nucleus to cytoplasm (Fig 1a). The tissues have noticeable nucleoli, other cells show multiple nucleoli, and multinucleated giant cells could be seen forming. A few of these monolayers displayed evidence of the proliferating, small, dark, overlapping layers. The observed monolayer growth of cells exhibited a minimal level of inhibition of growth when exposed to low concentrations (25 µg/ml). At this particular concentration, it was observed that the monolayer was disrupted, resulting in the loss of its confluent feature. Additionally, unique patchy inhibition was observed, accompanied by mild cellular swelling and vacuolated cytoplasm. Furthermore, some pyknotic cells were also found (Fig 1b).

**Table 4:** Human squamous cell carcinoma MTT assay

MTT						
Concentration µg/ml	24 h		48 h		72 h	
	Mean	SD	Mean	SD	Mean	SD
0	1.8489	0.00452	1.8632	0.00221	1.9232	0.0021
5	1.5865*	0.04657	1.2312*	0.012	1.1412*	0.0135
10	1.3286*	0.02835	1.0124*	0.028	1.0024*	0.0185
25	1.25565*	0.02547	0.8514*	0.0198	0.6522*	0.0233
50	1.03005*	0.08834	0.5421*	0.0125	0.4461*	0.0262
125	0.83903*	0.01519	0.4772*	0.01562	0.2872*	0.0100
250	0.53975*	0.01349	0.2142*	0.01447	0.1052*	0.0134
500	0.01884*	0.00059	0.0124*	0.00052	0.0013*	0.0006

p-value (concentration) = 0.000004; p-value (exposure time) = 0.012. Each number represents the mean of three replicates. *Significant at p<0.05.

**Table 5:** Inhibition rate of HSCC at different concentrations and different exposure times of MEHSP

MEHSP			
Concentration µg/ml	24 h IR%	48 h IR%	72 h IR%
0	0.0	0.0	0.0
5	14.192	33.920	40.66
10	28.141	45.663	47.87
25	32.086	54.304	66.08
50	44.288	70.904	76.80
125	54.623	74.388	85.066
250	70.807	88.503	94.529
500	98.983	99.334	99.932

**Table 6:** Viability rate of HSCC at different concentrations and different exposure times of MEHSP by Trypan blue assay

Trypan blue assay			
Concentration µg/ml	Viability rate %		
	24 h	48 h	72 h
0.0	98.23	98.54	98.62
5	87.25	79.45	77.44
10	72.15	68.12	63.52
25	66.32	56.47	51.65
50	52.78	42.17	36.12
125	43.12	38.16	32.14
250	22.17	18.19	16.12
500	10.89	9.21	8.31

**Fig 2:**
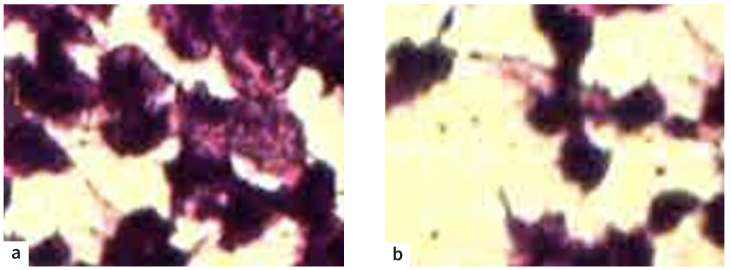
a. Exposure to 125 µg/ml methanol extract of *Hibiscus sabdariffa*. Patchy growth inhibition and disruption of confluent monolayer. b. Exposure to 250 µg/ml of methanol extract of *Hibiscus sabdariffa*. Significant loss of cellular characteristics, a high number of dead cells, and a high content of cellular debris are apparent. H&E stain; 400X magnification.

Higher concentrations of MEHSP (125 and 250 µg/ml) resulted in comparable alterations, characterised by increased patchy inhibition of cell growth and early phases of cytolysis. The dead cells became more obvious compared to those seen in low concentration. On the other hand, after treatment with the highest concentrations of MEHSP (125 and 250 µg/ml), the observed effects appeared to be more pronounced, with notable morphological characteristics indicating complete cell lysis accompanied by bare nuclei. Examining the cells revealed that few still displayed their normal characteristics and that there were mostly deceased cells exhibiting pyknosis, accompanied by debris from the cells (Fig 2).

### Cytogenetic Analysis

**Fig 3:**
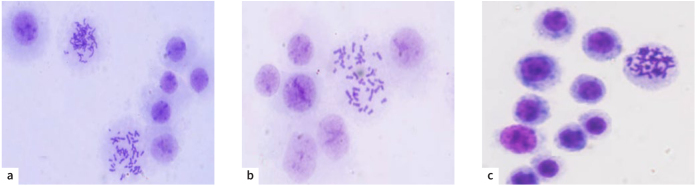
Peripheral blood lymphocytes. a. untreated (control); b. treated with 50 µg/ml of MEHSP; c. treated with 250 µg/ml of MEHSP. 400X magnification, Giemsa stain.

Data from the chromosomal study revealed a considerable increase in the peripheral blood lymphocytes’ (PBL) mitotic index (MI) and blastogenic index (BI) with low doses (0.0-25 µg/l). Genotoxicity – such as chromosomal breakages, translocations, and deletions – was absent, as were any harmful effects on human peripheral blood cells at the quantities used. At relatively high doses (50-500 µg/l), a decrease in cytogenetic indicators was observed, but the decrease was not statistically significant, which indicates a limited effect on somatic cells (Table 7; Fig 3).

**Table 7:** Cytogenetic assay of MEHSP at various concentrations of a healthy person’s blood lymphocytes

Concentration (µg/ml)	BI	MI	TCA
0.0	62.421 ± 0.44	1.85 ± 0.02	0.26 ± 0.01
5	65.11 ± 0.125	1.92 ± 0.01	0.25 ± 0.01
10	67.25 ± 0.33	2.11 ± 0.01	0.23 ± 0.01
25	61.15 ± 0.26	2.01 ± 0.03	0.0
50	57.55 ± 0.48	1.86 ± 0.06	0.0
125	55.18 ± 0.66	1.79 ± 0.05	0.0
250	52.33 ± 0.32	1.77 ± 0.03	0.0
500	51.29 ± 0.12	1.72 ± 0.01	0.0

Each number represents means ± SD for three replicates.

It was observed that the stem cells (blast cells) retained their ability to divide, even with treatment at concentrations of 50 µg/l and 250 µg/l, and did not lose their ability to transform into blast cells, in preparation to create new cells while preserving the chromatin without any mutations.

## Discussion

Epidemiological studies have firmly established the correlation between diet and the risk of developing cancer. There has been an increasing focus on edible plants as potential reservoirs of anticancer compounds. *Hibiscus sabdariffa* is a plant that is globally recognised for its medicinal properties. Anthocyanin compounds, polyphenols, and flavonoids are abundant in *H. sabdariffa* and have become popular due to their chemo-preventive properties. Different types of human cancerous cells, such as gastric, prostate, cervical, and mammary cancer cells, have demonstrated vulnerability to the cytotoxic impact of *H. sabdariffa*. Cell viability is an important criterion to evaluate the cytotoxic effect of a plant extract. Decreased cell viability with increasing concentrations indicates the potency of the particular extract. This study investigated the cytotoxic effect of MEHSP on the HSCC cell line, finding a statistically significant cytotoxic potential of MEHSP in a dose- and time-dependent manner. According to Laskar and Mazumder,^15^ the cytotoxic effect of *H. sabdariffa* is ascribed to its capacity to modulate crucial pro-oncogenic and anti-oncogenic paths, encompassing selective cytotoxicity, induction of apoptosis, inhibition of metastasis, and suppression of angiogenesis. In addition, it has been proposed that including *H. sabdariffa* in chemotherapeutic drugs (e.g., cisplatin and taxol) may enhance the initiation of apoptosis. This is achieved by augmenting oxidative stress and reducing mitochondrial membrane potential, for instance, in triple-negative breast cancer cells, compared to treatment with the drugs alone.^27^ Moreover, our results have shown statistically significant antioxidant activity of MEHSP that increased with increasing concentrations. The key constituents of *H. sabdariffa* that contribute to its pharmacological efficacy encompass flavonoids, organic acids, anthocyanins, and polysaccharides.^17^ There is a frequently observed association between chronic inflammation, stress, and the development of chronic diseases, such as cancer.^13^ The processes mentioned above play a role in the progression of cancer, including cell alteration, growth, vascular development, metastases, and survival.^1,16^ As previously studied, *H. sabdariffa* possesses a high concentration of various polyphenols, which exhibit strong antioxidant properties. Furthermore, anthocyanins possess antioxidant properties and play a role in mediating physiological processes associated with the suppression of cancer.^17^ The botanical source and extraction methodology significantly influence the volatile structure of the extract, consequently influencing the appropriate dosage.^10^ For instance, compared to ethanol-based extracts of the leaves or aqueous extracts of other plant parts,^26^ greater antioxicant activitiy was achieved with an ethanol extract of calyx components.^10^

The results of our study are promising concerning the anticancer properties of *Hibiscus sabdariffa*. However, some limitations stem from insufficient data on the optimal dosage needed to elicit the desired physiological responses in humans. Therefore, it is suggested that a suitable clinical setting could support the recommendation of *H. sabdariffa* as a supplementary treatment for cancer patients.

## Conclusion

This study demonstrated that *Hibiscus sabdariffa* has cytotoxic and antioxidant properties against HSCC. Thus, *Hibiscus sabdariffa* possesses potent anticancer properties that are underexplored as potential candidates for use in chemotherapy.
